# Factors influencing rapid antiretroviral therapy initiation in Jiulongpo, Chongqing, China: a retrospective cohort from 2018 to 2022

**DOI:** 10.1186/s12981-024-00601-y

**Published:** 2024-03-17

**Authors:** Cheng Chen, Hao Chen, Lingli Wu, Qin Gong, Jingchun He

**Affiliations:** Center for Disease Control and Prevention of Jiulongpo Distract, Chongqing, China

**Keywords:** Antiretroviral therapy, Rapid antiretroviral therapy, Rapid ART initiation, HIV, AIDS, CD4

## Abstract

**Background:**

Antiretroviral Therapy (ART) is pivotal in extending the lives of people living with HIV (PLWH) and minimizing transmission. Rapid ART initiation, defined as commencing ART within seven days of HIV diagnosis, is recommended for all PLWH.

**Method:**

A retrospective cohort study was conducted using data from the China Information System for Disease Control and Prevention. This study included PLWH diagnosed between January 2018 and December 2021 and treated by December 2022. Factors influencing rapid ART initiation were examined using univariate and multivariate Cox regression analyses.

**Results:**

The study analyzed 1310 cases. The majority were male (77.4%), over 50 years old (46.7%), and contracted HIV through heterosexual transmission (70.0%). Rapid ART initiation was observed in 36.6% (*n* = 479) of cases, with a cumulative treatment rate of 72.9% within 30 days post-diagnosis. Heterosexual contact was associated with longer intervals from diagnosis to treatment initiation compared to homosexual contact (Adjusted Hazard Ratio (HR) = 0.813, 95% Confidence Interval (CI): 0.668–0.988). Individuals older than 50 years (Adjusted HR = 1.852, 95%CI: 1.149–2.985) were more likely to initiate ART rapidly. Conversely, treatment at the Second Public Hospital (Adjusted HR = 0.483, 95% CI: 0.330–0.708) and a CD4 cell counts above 500 (Adjusted HR = 0.553, 95% CI: 0.332–0.921) were associated with a lower likelihood of initiating treatment within seven days.

**Conclusions:**

A higher CD4 cell counts and receiving care in local public hospitals may deter rapid ART initiation. Providing CD4 counts results at diagnosis and offering testing and treatment in the same facility could enhance the rate of rapid ART initiation.

HIV continues to be a major cause of mortality globally. As of 2021, there were approximately 38.4 million people living with HIV (PLWH) across the world, with about 1.5 million new infections occurring that year, a significant decrease from the 3.2 million new infections recorded in 1996 [1]. HIV-related fatalities reached their zenith in 2004 with 1.8 million deaths [2] but have since declined to 0.65 million in 2021 [1]. The reduction in mortality rates is largely attributed to the progressive improvements in antiretroviral therapy (ART), both in terms of drug development and accessibility, which has also played a crucial role in preventing new HIV infections.

The criteria for initiating ART have evolved over time, influenced by the availability of ART and the emergence of new research evidence. Initially, ART was recommended for PLWH based on their CD4 cell levels [3]. By 2015, guidelines had shifted to recommend ART for all individuals diagnosed with HIV, regardless of CD4 counts [4]. In 2016, the World Health Organization (WHO) advanced this approach by introducing a “treat all” policy, advocating for ART for all PLWH irrespective of their CD4 cell counts or clinical symptoms. Transmission of HIV may occur during the interval from diagnosis to treatment, and previous evidence also showed that earlier use of ART resulted in better clinical outcomes of people living with HIV compared with delayed treatment. Consequently, WHO, in 2017, recommended rapid ART initiation, defined as commencing ART within seven days of HIV diagnosis [5]. WHO advises that rapid ART initiation should be available to all PLWH after a confirmed HIV diagnosis and clinical assessment, and should be offered on the same day for those prepared to begin treatment [5].

Rapid initiation of ART offers significant clinical and public health advantages [6–8]. Previous randomized trials have demonstrated that the likelihood of initiating ART within 90 days and within 12 months is enhanced by the implementation of rapid ART initiation strategies [9]. This approach is effective in addressing patient concerns [10], shortening the duration to achieve viral suppression [11, 12], and reducing the incidence of tuberculosis and severe bacterial infections [13]. In Zambia, rapid initiation of ART significantly increased the proportion of patients commencing ART on the same day as enrollment, resulting in modest improvements in retention in care under real-world conditions [14]. Epidemiological and economic analyses in Spain revealed that rapid ART initiation could prevent up to 992 potential HIV infections and yield savings of approximately €323 million over the next two decades [15]. In the Ekurhuleni District of South Africa, a retrospective cohort study reported that 54% of PLWH initiated ART within seven days of diagnosis confirmation [16]. A citywide rapid ART initiative in San Francisco reduced the median time from diagnosis to care from seven to two days [17]. Furthermore, rapid ART initiation has been implemented in community settings and outside traditional health facilities [18].

Guidelines for rapid ART initiation have been issued in the USA, Europe, and several countries in the Asia-Pacific region [19]. In 2018, Chinese treatment guidelines recommended ART for all individuals living with HIV, regardless of CD4 counts, with a particular emphasis on rapid and same-day ART initiation in the 2021 update [20, 21]. To halt the HIV epidemic by 2030, the Joint United Nations Programme on HIV/AIDS (UNAIDS) has set a revised treatment target of 95-95-95 by 2030 [22]. Numerous strategies have been developed to minimize the pre-ART period, necessitating the simplification of ART delivery to expand coverage [23, 24]. Yet, the real-world characteristics of rapid ART initiation and its influencing factors remain underexplored. Investigating these factors is crucial for achieving the 95% treatment target and preventing potential HIV infections. Hence, we conducted a retrospective cohort study to examine the characteristics of rapid ART initiation and identify factors influencing the timing of ART initiation.

## Methods

### Study setting

This observational study analyzed a retrospective cohort of patients diagnosed with HIV from January 2018 to December 2021. Data were sourced from the China Information System for Disease Control and Prevention, which catalogs diagnostic and treatment information as reported by agencies and designated treatment hospitals. Authorized health workers inputted information on PLWH, including transmission mode, diagnosis time, and confirmatory test results. Upon receiving a positive HIV confirmatory test, reporting agency personnel recorded the PLWH’s personal information. Physicians at designated hospitals entered physical examination outcomes, treatment initiation dates, and drug regimens into the system. The study encompassed three medical facilities, all government-designated for HIV treatment. The Chongqing Public Health Medical Center (CMC) serves as a major infectious disease hospital, treating HIV-infected patients from across Chongqing. Additionally, two public hospitals in Jiulongpo District, The First Public Hospital in Jiulongpo (FPH) and The Second Public Hospital in Jiulongpo (SPH), provide treatment exclusively for local residents.

### Patient population

This study focused on patients registered in the information system for HIV diagnosis and treatment. The inclusion criteria were: (1) residence in Jiulongpo District, Chongqing; (2) diagnosis between January 2018 and December 2021; (3) initiation of treatment before December 2022. Exclusion criteria included: (1) absence of treatment initiation date; (2) lack of CD4 test results; (3) conditions contraindicating rapid treatment, including pulmonary tuberculosis, extrapulmonary tuberculosis, Mycobacterium avium complex infection, and cryptococcal meningitis. The observational period commenced on the diagnosis date and concluded on the antiviral therapy initiation date. Collected data encompassed demographic and clinical information, including sex, age, educational level, marital status, reporting agency, initial treatment facility, treatment initiation date, CD4 cell counts at care entry, baseline alanine aminotransferase (ALT) levels, HIV-related diseases, and history of sexually transmitted diseases. Treatment intervals of ART was defined as the number of days between the first-ever HIV diagnosis date and the ART start date recorded in the system Rapid ART initiation was defined as initiating ART within seven days of HIV diagnosis [10]. Treatment intervals of 8–30 days were categorized as Early ART, 31 days to 1 year as Intermediate ART, and more than 1 year as Delayed ART in this study [25].

### Analysis methods

The count data were summarized by the number of cases and their respective percentages, and the chi-square test facilitated group comparisons. Survival analysis has the advantage to take both outcome and the time interval into account. The Kaplan–Meier survival curve was used to investigate differences in treatment initiation across age groups, CD4 cell counts, and initial treatment facilities. Both univariate and multivariate Cox regression analyses were employed to examine factors influencing ART and rapid ART initiation. In the multivariate Cox regression, all variables were included to calculate the adjusted hazard ratios (HR) and their 95% CI. Statistical analysis was conducted using a two-tailed test, with a p-value of less than 0.05 denoting statistical significance. Data analysis was performed using R version 4.2.1.

## Results

### Demographic factors

Between 2018 and 2021, a total of 1533 PLWH were diagnosed, of which 1324 initiated ART before December 2022. Five individuals were unable to obtain CD4 results. Nine cases exhibited contraindications for rapid treatment, including six instances of pulmonary tuberculosis, two of extrapulmonary tuberculosis, and one of Mycobacterium avium complex infection. Consequently, 1310 cases were eligible for analysis in this study. The majority of PLWH analyzed were male (77.4%, *n* = 1014), aged 50 and above (46.7%, *n* = 612), and married (48.5%, *n* = 636). Homosexual transmission constituted 28.5% of cases, while heterosexual transmission accounted for 70.0%.

## Diagnosis and treatment

Between 2018 and 2021, annually more than 300 PLWH were reported, with 49.8% identified by the Centers for Disease Control and Prevention (CDC) and 50.2% by hospitals. The rate of rapid ART initiation was 36.5% (*n* = 479), and the cumulative treatment initiation rate within 30 days post-diagnosis reached 73.0%. Notably, the rate of rapid ART initiation increased from 22.7% in 2018 to 44.9% in 2021.

Among these, 30.8% of PLWH received their initial treatment at CMC, 40.3% at the FPH, and 24.3% at the SPH. The rates of rapid ART initiation were 51.9% at CMC, 30.7% at FPH, and 26.7% at SPH, showing statistically significant differences (*P* < 0.001) (See Table 1).

**Table 1:** Characteristics of patients and of each group based on antiretroviral therapy initiation after confirmed HIV diagnosis

Variables	Total *N* = 1310(%)	ART initiation after confirmed HIV diagnosis				χ^2^	p
		Rapid ≤ 7 day N = *N* = 479	Early 8–30 days *N* = 477 (%)	Intermediate 31 days-1 year *N* = 309 (%)	Delayed >1 year *N* = 45(%)		
**Sex**							
Male	1014 (77.4)	372 (36.7)	369 (36.4)	237 (23.4)	36 (3.6)	0.280	0.964
Female	296 (22.6)	107 (36.1)	108 (36.5)	72 (24.3)	9 (3.0)		
**Age (years)**							
10–29	301 (23.0)	100 (33.2)	108 (35.9)	77(25.6)	16(5.3)	8.384	0.211
30–49	397 (30.3)	155 (39.0)	139 (35.0)	95 (23.9)	8 (2.0)		
50–86	612 (46.7)	224 (36.6)	230 (37.6)	137 (22.4)	21 (3.4)		
**Education**							
Primary and illiteracy	367 (28.0)	127 (34.6)	149 (40.6)	78 (21.3)	13 (3.5)	9.548	0.388
Middle school	357 (27.3)	132(37.0)	122 (34.2)	91 (25.5)	12 (3.4)		
High School	259 (19.8)	86 (33.2)	99 (38.2)	67 (25.9)	7 (2.7)		
Tertiary	327 (25.0)	134 (41.0)	107 (32.7)	73 (22.3)	13 (28.9)		
**Marital status**							
Married	636 (48.5)	241 (37.9)	235 (36.9)	144 (22.6)	16 (2.5)	8.385	0.211
Divorced	225 (17.2)	69 (30.7)	88 (39.1)	60 (26.7)	8 (3.6)		
Single	449 (34.3)	169 (37.6)	154 (34.3)	105 (23.4)	21 (4.7)		
**Transmission mode**							
Homosexual contact	373 (28.5)	147 (39.4)	134 (35.9)	78 (20.9)	14 (3.8)	6.240	0.397
Heterosexual contact	917 (70.0)	327 (35.7)	336 (36.6)	223 (24.3)	31 (3.4)		
Others	20 (1.5)	5 (25.0)	7 (35.0)	8 (40.0)	0 (0)		
**Year diagnosed**							
2018	321 (24.5)	73(22.7)	123 (38.3)	103 (32.1)	22 (6.9)	72.597	0.001**
2019	339 (25.9)	144 (42.5)	121 (35.7)	60 (17.7)	14 (4.1)		
2020	325 (24.8)	116 (35.7)	110 (33.8)	91 (28.0)	8 (2.5)		
2021	325 (24.8)	146 (44.9)	123 (37.8)	55 (16.9)	1 (0.3)		
**Reporting agency**							
Centers for Disease Control and Prevention	652 (49.8)	219 (33.6)	258 (39.6)	149 (22.9)	26 (4.0)	8.151	0.043*
Hospitals	658 (50.2)	260(39.5)	219 (33.3)	160 (24.3)	19 (2.9)		
**First-time treatment facility**							
Chongqing Public Health Medical Center	403 (30.8)	209 (51.9)	131 (32.5)	56 (13.0)	7 (1.7)	72.588	0.001**
The First Public Hospital in Jiulongpo	528 (40.3)	162 (30.7)	200 (37.9)	144 (27.3)	22 (4.2)		
The Second Public Hospital in Jiulongpo	318 (24.3)	85 (26.7)	129 (40.6)	90 (28.3)	14 (4.4)		
Others	61 (4.7)	23 (37.7)	17 (27.9)	19 (31.1)	2 (3.3)		
**History of sexually transmitted diseases**							
No	984 (75.1)	345 (35.1)	374 (38.0)	235 (23.9)	30 (3.0)	15.136	0.019*
Yes	137 (10.5)	68 (49.6)	39 (28.5)	24 (17.5)	6 (4.4)		
Unknown	189 (14.4)	66 (34.9)	64 (33.9)	50 (26.5)	9 (4.8)		
**CD4 cell counts cells/µL**							
< 200	632 (48.2)	272 (43.0)	233 (36.9)	122 (19.3)	5 (0.8)	63.027	0.001**
200–500	582 (44.4)	183 (31.4)	216 (37.1)	154 (26.5)	29 (5.0)		
> 500	96 (7.3)	24 (25.0)	28 (29.2)	33 (34.4)	11 (11.5)		
**ALT abnormalities**							
No	647 (64.2)	216 (33.4)	231 (35.7)	173 (26.7)	27 (4.2)	3.603	0.308
Yes	361 (35.8)	108 (29.9)	150 (41.6)	91 (25.2)	12 (3.3)		

*p < 0.05

**p < 0.001

**Fig. 1:**
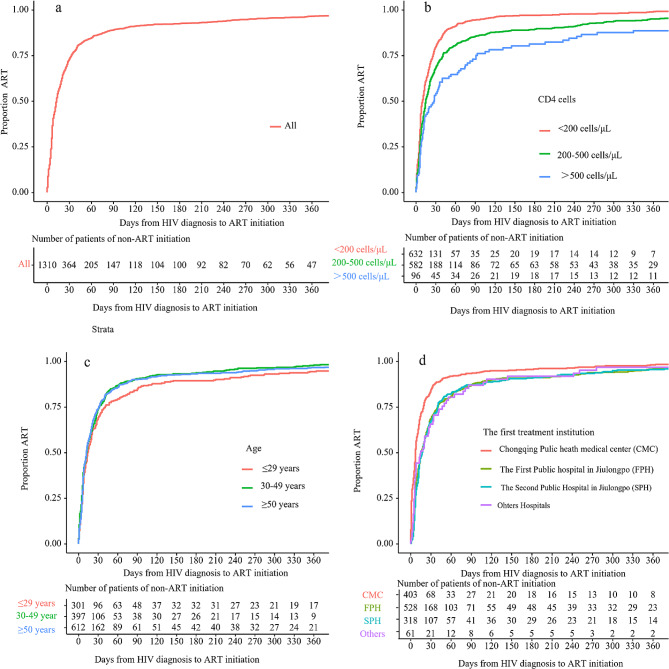
Kaplan-Meier plots of cumulative ART initiation event: (**a**) overall; (**b**) group by CD4 cells; (**c**) group by ages; (**d**) group by the first treatment initiation

The median time to the initiation of treatment across the study population was 13 days, with a 95% confidence interval (CI) of 12 to 14 days, as shown in Fig. 1a. For individuals with CD4 counts less than 200 cells/µL, the median time to treatment initiation was 10 days, 95% CI 8 to 12 days. For those with CD4 counts between 200 and 500 cells/µL, it was 15 days, 95% CI 14 to 18 days. Participants with CD4 counts of 500 cells/µL or higher had a median initiation time of 27.5 days, 95% CI 13 to 40 days, as depicted in Fig. 1b.

At the CMC, the median time to treatment initiation was 7 days, with a 95% CI of 7 to 8 days. At the SPH, it was longer, at 17 days, with a 95% CI of 14 to 20 days, as illustrated in Fig. 1d.

## Influencing factors associated with time of ART initiation

The univariate analysis indicated that age, diagnosis year, reporting agency, first-time treatment facility, history of sexually transmitted diseases, and CD4 cell counts significantly impacted ART initiation, with all differences being statistically significant (*p* < 0.05), as illustrated in Table 2. Compared to individuals younger than 30 years, those aged between 30 and 49 years (HR = 1.233, 95% CI: 1.060 to 1.434), and those older than 50 years (HR = 1.198, 95% CI: 1.042 to 1.377) had a shorter time from diagnosis to ART initiation.

Multivariate Cox regression analysis determined that transmission mode, diagnosis year, reporting agency, first-time treatment facility, and CD4 cell counts were influential in determining the timing of ART initiation. Patients with heterosexual contacts experienced longer delays from diagnosis to ART initiation compared to those with homosexual contacts (Adjusted HR = 0.813, 95%CI: 0.668 to 0.988). Relative to participants with CD4 counts below 200 cells/µL, individuals with CD4 counts between 200 and 500 cells/µL (Adjusted HR = 0.743, 95% CI: 0.647 to 0.852), and those with more than 500 cells/µL (Adjusted HR = 0.591, 95% CI: 0.455 to 0.767) faced longer intervals from diagnosis to ART commencement.

**Table 2:** Univariate and multivariate cox regression analysis for factors influencing ART initiation in HIV/AIDS cases in Jiulongpo district from 2018 to 2021

Variables	Univariate		Multivariate	
	HR (95%CI)	*P*	Adjusted HR (95%CI)	*P*
**Sex**				
Male				
Female	1.014 (0.891~1.154)	0.835	0.999 (0.788~1.266)	0.991
**Age (years)**				
10–29				
30–49	1.233 (1.060~1.434)	0.007*	1.217 (0.973~1.522)	0.085
50–86	1.198 (1.042~1.377)	0.011*	1.236 (0.950~1.606)	0.114
**Education**				
Primary and illiteracy				
Middle school	0.998 (0.911~1.094)	0.974	0.948 (0.797~1.127)	0.543
High School	0.975 (0.881~1.08)	0.629	0.916 (0.747~1.122)	0.396
Tertiary	0.995 (0.905~1.093)	0.912	1.073 (0.847~1.36)	0.560
**Marital status**				
Married				
Divorced	0.905 (0.777~1.054)	0.199	0.937 (0.786~1.116)	0.465
Singe	0.893 (0.791~1.009)	0.069	0.810 (0.650~1.010)	0.061
**Transmission mode**				
Homosexual contact				
Heterosexual contact	0.989 (0.876~1.116)	0.852	0.813 (0.668~0.988)	0.038*
Others	0.785 (0.500~1.232)	0.293	0.669 (0.372~1.203)	0.179
**Year diagnosed**				
2018				
2019	1.386 (1.189~1.615)	< 0.001**	1.17 (0.972~1.408)	0.096
2020	1.297 (1.111~1.515)	0.001*	1.133 (0.941~1.366)	0.188
2021	1.756 (1.502~2.055)	< 0.001**	1.751 (1.447~2.119)	< 0.001**
**Reporting Units**				
Hospitals				
Centers for Disease Control and Prevention	0.924 (0.829~1.029)	0.151	1.195 (1.029~1.388)	0.020*
**First-time treatment facility**				
Chongqing Public Health Medical Center				
The First Public Hospital in Jiulongpo	0.626 (0.549~0.713)	< 0.001**	0.936 (0.757~1.157)	0.540
The Second Public Hospital in Jiulongpo	0.596 (0.514~0.691)	< 0.001**	0.781 (0.621~0.981)	0.034*
Others	0.637 (0.486~0.834)	0.001*	1.551 (0.997~2.411)	0.052
**History of sexually transmitted diseases**				
Yes				
No	0.798 (0.667~0.955)	0.014*	0.885 (0.715~1.095)	0.259
Unknown	0.750 (0.602~0.935)	0.011*	0.870 (0.659~1.149)	0.326
**CD4 cells counts cells/µL**				
< 200				
200–500	0.700 (0.624~0.785)	< 0.001**	0.743 (0.647~0.852)	< 0.001**
≥ 500	0.500 (0.401~0.623)	< 0.001**	0.591 (0.455~0.767)	< 0.001**
**ALT abnormalities**				
No				
Yes	1.050 (0.923~1.194)	0.460	1.024 (0.835~1.256)	0.819

*p < 0.05

**p < 0.001

### Influencing factors associated with rapid ART

The univariate analysis results indicated that the year of diagnosis, reporting agency, First-time treatment facility, history of sexually transmitted diseases, and CD4 cell count significantly impacted the likelihood of rapid ART initiation, as evidenced by statistical significance (*p* < 0.05) in Table 3. Multivariate analysis revealed that individuals over 50 years old had a higher propensity (HR = 1.852, 95% CI: 1.149–2.985) to initiate treatment within 7 days compared to those under 30 years of age. Patients whose first treatment facility was either the First Public Hospital in Jiulongpo (Adjusted HR = 0.663, 95% CI: 0.471–0.932) or the Second Public Hospital in Jiulongpo (Adjusted HR = 0.483, 95% CI: 0.330–0.708) exhibited a lower likelihood of initiating treatment within 7 days than those treated at CMC. Furthermore, compared to patients with CD4 counts below 200 cells/µL, individuals with CD4 counts between 200 and 500 cells/µL (Adjusted HR = 0.657, 95% CI: 0.518–0.834) and above 500 cells/µL (Adjusted HR = 0.553, 95% CI: 0.332–0.921) were less likely to initiate treatment within 7 days.

**Table 3:** Univariate and multivariate Cox regression analysis for factors influencing Rapid ART in HIV/AIDS cases in Jiulongpo district from 2018 to 2021

Variables	Univariate		Multivariate	
	HR (95%CI)	*P*	Adjusted HR (95%CI)	*P*
**Sex**				
Male				
Female	0.986 (0.795~1.222)	0.895	1.507 (0.961~2.362)	0.074
**Age (years)**				
10–29				
30–49	1.223 (0.951~1.573)	0.116	1.473 (0.989~2.192)	0.057
50–86	1.109 (0.876~1.404)	0.389	1.852 (1.149~2.985)	0.011*
**Education**				
Primary and illiteracy				
Middle school	1.087 (0.852~1.387)	0.500	1.050 (0.778~1.418)	0.750
High School	0.989 (0.752~1.300)	0.935	1.044 (0.726~1.502)	0.817
Tertiary	1.249 (0.980~1.593)	0.072	1.519 (1.004~2.296)	0.048*
**Marital status**				
Married				
Divorced	0.787 (0.602~1.029)	0.080	0.844 (0.616~1.155)	0.289
Singe	1.010 (0.830~1.229)	0.923	0.915 (0.615~1.363)	0.663
**Transmission mode**				
Homosexual contact				
Heterosexual contact	0.884 (0.728~1.074)	0.216	0.729 (0.511~1.040)	0.081
Others	0.567 (0.232~1.382)	0.212	0.635 (0.222~1.816)	0.397
Year diagnosed				
2018				
2019	2.072 (1.563~2.746)	< 0.001**	2.159 (1.501~3.107)	< 0.001**
2020	1.738 (1.297~2.330)	< 0.001**	1.483 (1.000~2.201)	0.05
2021	2.271 (1.715~3.008)	< 0.001**	2.369 (1.637~3.429)	< 0.001**
**Reporting agency**				
Hospitals				
Centers for Disease Control and Prevention	0.774 (0.646~0.926)	0.005*	1.228 (0.930,1.622)	0.148
**First-time treatment facility**				
Chongqing Public Health Medical Center				
The First Public Hospital in Jiulongpo	0.465 (0.379~0.571)	< 0.001**	0.663 (0.471~0.932)	0.018*
The Second Public Hospital in Jiulongpo	0.391 (0.304~0.503)	< 0.001**	0.483 (0.330~0.708)	< 0.001**
Others	0.604 (0.392~0.929)	0.022*	1.398 (0.715~2.733)	0.327
**History of sexually transmitted diseases**				
Yes				
No	0.614 (0.473~0.796)	< 0.001**	0.753 (0.541,1.048)	0.092
Unknown	0.624 (0.445~0.875)	0.006*	0.727 (0.453,1.165)	0.185
**CD4 cells counts cells/µL**				
< 200				
200–500	0.689 (0.571~0.831)	< 0.001**	0.657 (0.518~0.834)	0.001*
≥ 500	0.522 (0.344~0.793)	0.002*	0.553 (0.332~0.921)	0.023*
**ALT abnormalities**				
No				
Yes	0.884 (0.701~1.113)	0.293	0.703 (0.477~1.034)	0.074

*p < 0.05

**p < 0.001

## Characteristics of untreated PLWH

Among the 1533 patients infected, 209 did not commence antiretroviral therapy (ART) prior to 2022. Regarding gender distribution, no significant difference was observed between patients in ART and non-ART groups, as indicated in Table 4. Individuals of older age, lowereducational level, and those acquiring the infection through heterosexual transmission were less likely to initiate treatment. These disparities were statistically significant, with a *p*-value less than 0.001.

**Table 4:** Characteristics of people living with HIV diagnosed from January 2018 to December 2021

Variables	Total *N* = 1533	Non-ART *n* = 209	ART *n* = 1324	c^2^	*p*
**Sex**					
Male	1190 (77.6)	164(13.8)	1026 (86.2)	0.099	0.753
Female	343 (22.3)	45 (13.1)	298 (86.9)		
**Age (years)**					
10–29	323 (21.1)	20 (6.2)	303 (93.8)	23.323	< 0.001**
30–49	459 (29.9)	60 (13.1)	399 (86.9)		
50–86	751 (49.0)	129 (17.2)	622 (82.8)		
**Education**					
Primary and illiteracy	464 (30.3)	90 (19.4)	374 (80.6)	30.336	< 0.001**
Middle school	423 (27.6)	62 (14.7)	361 (85.3)		
High School	296 (19.3)	35 (11.8)	261 (88.2)		
Tertiary	350 (22.8)	22 (6.3)	328 (93.7)		
**Transmission mode**					
Heterosexual contact	1104 (72.0)	176 (15.9)	928 (84.1)	19.413	< 0.001**
Homosexual contact	404 (26.4)	29 (7.2)	375 (92.8)		
Others	25 (1.6)	4 (16.0)	21 (84.0)		

** *P* < 0.001

## Discussion

The prevalence of rapid ART initiation increased from 22.7% in 2018 to 44.9% in 2021, aligning with the 32.7% observed in Shenyang, China [26]. In metropolitan Taipei, Taiwan, rapid ART initiation reached 68.3% in 2017 [27], while same-day initiation (SDI) achieved a rate of 69.2% in KwaZulu Natal, South Africa, in 2020 [28]. Following the implementation of the “treat all” policy, there has been a noticeable trend towards a reduction in the median time to ART initiation post-HIV infection screening [29]. However, in Jiulongpo, Chongqing, the rate of rapid treatment initiation still requires enhancement through various strategies. The information system recorded only a limited number of patients with opportunistic infections, suggesting the possible existence of additional cases unsuitable for rapid ART initiation. Therefore, caution is advised when making generalizations about the rapid treatment initiation rate.

Perceived healthiness may deter rapid initiation of ART. Data show that patients with a CD4 counts > 500 cells/mm³ were less likely to initiate ART within 7 days. This finding aligns with previous research [25]. Across varying threshold values, the linkage to care for PLWH with higher CD4 counts occurred later compared to those with lower counts, e.g., CD4 < 100 cells/mm³ [25], CD4 < 200 cells/mm³ [30]. In the asymptomatic stages of HIV, marked by elevated CD4 levels, PLWH may feel well and not perceive themselves as infected. Accepting one’s infection status can be challenging for some, particularly those with low perceived risk of HIV, leading to a lower likelihood of initiating ART. Moreover, among patients with high CD4 counts, repeat HIV testing and the anticipation of ART side effects also contributed to treatment initiation delays [31]. In primary care settings, the CD4 counts can serve as a vital tool for health promotion. Informing patients of their CD4 results at the time of diagnosis, rather than afterwards, can aid in understanding the virus’s impact on their immunity. Recognizing the disease’s presence can underscore the need and urgency for treatment. In Jiulongpo District, the implementation of simultaneous confirmatory and CD4 testing since 2021 may account for observed year-to-year differences.

Elderly patients may have been considered at higher risk for adverse health outcomes associated with HIV and, consequently, initiated ART earlier than their younger counterparts, who might have been perceived as healthier [25]. Young individuals may fear family discovery of their HIV status through observation of medication intake. Furthermore, young adults often require time away from work for medical appointments, and the potential loss of income due to missed work could postpone their decision to commence ART [32].

In Croatia, the median duration from HIV diagnosis to ART initiation among man who have sex with man (MSM) was 1.1 months, longer than the 0.6 months observed in individuals with heterosexual contacts [25]. Contrarily, in Jiulongpo, Chongqing, MSM were found to initiate ART in a shorter timeframe compared to heterosexual individuals. MSM in China may have greater access to HIV testing and treatment services via social media platforms, such as WeChat and Blued. WeChat, with its 900 million active users in China, and MSM key opinion leaders on the platform, offer advertisements for HIV testing programs and distribute free HIV test kits to MSM [33]. Blued, a Beijing-based media company, operates a dating application popular among Chinese MSM and offers social services, including drugs for pre-exposure prophylaxis, on personal homepages [34]. Additionally, MSM can receive support from non-governmental organizations (NGOs) during the HIV testing process [35] and in making treatment decisions. A prior study revealed that MSM were more likely to connect with ART clinics through NGOs rather than visiting the closest ART clinic [36]. Integrating support services for rapid ART initiation into the tasks of NGOs could be a critical strategy to reduce the time from diagnosis to ART initiation in the future.

Individuals diagnosed at CDC experienced a shorter duration from diagnosis to treatment initiation compared to those diagnosed in hospitals. A contributing factor may be the availability of directive counseling and referrals at CDC facilities. In Chongqing’s CDC, there is typically a designated HIV counseling room with staff specialized in HIV counseling, and the CDC is tasked with enhancing the antiretroviral treatment uptake within its jurisdiction. Prior research has demonstrated that supportive-directive counseling significantly influences patients’ decisions to initiate ART, their adherence to treatment, and the adoption of numerous lifestyle modifications [37]. In contrast, in hospitals, physicians across various clinical specialties convey the diagnosis to the infected individuals, and a general lack of awareness about rapid ART initiation among physicians could result in PLWH not prioritizing ART as essential. The absence of a statistically significant difference in rapid treatment initiation between hospitals and CDC indicates that barriers still exist in both settings that prolong the time from diagnosis to ART initiation.

The choice of first-time treatment facility significantly impacted the timeliness of treatment initiation within 7 days post-HIV diagnosis. Our findings indicated that the interval between HIV diagnosis and initiation of treatment was shorter for PLWH receiving care at CMC, in contrast to longer intervals observed for those treated at two designated medical institutions in Jiulongpo District. CMC, as a designated treatment center, offers both diagnostic and treatment services at a single location for all PLWH residing in Chongqing. However, the two public hospitals in Jiulongpo District provide only treatment services, with diagnoses typically made at other facilities, including Centers for Disease Control and Prevention or hospitals equipped for diagnosis. Regarding service accessibility, CMC offers diagnostic and treatment services on weekdays and weekends, whereas the two public hospitals in Jiulongpo District limit treatment to weekdays. Rapid service provision is particularly beneficial for younger patients, minimizing time away from employment. The lack of sufficient HIV clinic medical providers has also been identified as a significant barrier to rapid ART initiation in the Southern United States [33]. Co-locating diagnostic and treatment services within the same facility may expedite treatment initiation, necessitating the gradual enhancement of diagnostic capabilities in existing designated hospitals. Future research should prioritize improving ART processes and services in these hospitals. While most PLWH in Jiulongpo District received treatment at the three hospitals discussed, a minority independently procured medications, with their information unrecorded in the system, thus precluding analysis of treatment initiation timing among this group.

## Conclusions

Rapid ART initiation in Jiulongpo, Chongqing, still faces several impediments. Factors such as higher CD4 cell counts, age under 30 years, heterosexual contact, and inadequate medical care contribute to lower likelihoods of rapid ART initiation. Simultaneous disclosure of CD4 counts results at diagnosis, coupled with offering testing and treatment within the same medical facility, could enhance the rate of rapid ART initiation. Furthermore, increased support services regarding rapid ART initiation from non-governmental organizations represent a crucial health promotion strategy moving forward.
